# MET异常NSCLC诊疗专家共识（2025版）

**DOI:** 10.3779/j.issn.1009-3419.2025.102.01

**Published:** 2025-03-21

**Authors:** Jun CHEN, Baohui HAN, Yi HU, Jian HU, Lung Cancer Specialty Committee of Chinese Elderly Health Care Association

**Keywords:** 肺肿瘤, MET异常, MET抑制剂, 治疗, Lung neoplasms, MET abnormalities, MET inhibitors, Treatment

## Abstract

间质上皮细胞转化因子（mesenchymal-epithelial transition factor, MET）基因位于人类7号染色体上，对细胞的增殖、迁移、侵袭和血管生成等生理过程起着至关重要的调控作用。MET基因是非小细胞肺癌（non-small cell lung cancer, NSCLC）的重要驱动基因之一，其异常形式多样，包括MET 14号外显子（MET exon 14, METex14）跳跃突变、MET基因扩增、MET融合、MET蛋白过表达以及MET活化突变等。随着对MET异常机制的深入理解，针对这些异常的治疗策略日益受到重视，且多项研究已证实携带MET异常的NSCLC患者能从这些治疗中显著获益。中国老年保健协会肺癌专业委员会致力于协助中国医师的临床实践，并为临床工作提供规范化指导，因此组织各专家委员结合临床实践经验并探寻循证医学证据，针对目前MET异常NSCLC诊断和治疗的临床问题给予专业建议，并制定专家共识《MET异常NSCLC诊疗专家共识（2025版）》，以期优化患者的治疗结果。

## Abstract (en)

The mesenchymal-epithelial transition factor (MET) gene, located on human chromosome 7, plays a crucial role in the regulation of physiological processes such as cell proliferation, migration, invasion, and angiogenesis. The MET gene is one of the key drivers in non-small cell lung cancer (NSCLC), with various forms of abnormalities including MET exon 14 (METex14) skipping mutations, MET gene amplification, MET fusions, MET protein overexpression, MET activating mutations and etc. With an increasing understanding of the mechanisms underlying MET abnormalities, therapeutic strategies targeting these abnormalities have gained significant attention, and numerous studies have confirmed that NSCLC patients with MET abnormalities can derive substantial benefits from such treatments. Lung Cancer Specialty Committee of Chinese Elderly Health Care Association organized a panel of experts to provide professional recommendations on current clinical issues in the diagnosis and treatment of MET-aberrant NSCLC, combining clinical practice experiences and evidence-based medical evidences. The "Expert Consensus on Diagnosis and Treatment of NSCLC with MET Abnormalities (2025 Version)" has been formulated to provide standardized guidances for clinical practice in China, with the aim of optimizing the treatment outcomes.

非小细胞肺癌（non-small cell lung cancer, NSCLC）是肺癌中最常见的病理类型。近二十年来，针对NSCLC的靶向治疗研究取得了显著进展，并极大地延长了患者的生存期。在众多NSCLC的肿瘤驱动基因中，间质上皮细胞转化因子（mesenchymal-epithelial transition factor, MET）基因亦属于关键之一，其被认为是继表皮生长因子受体（epidermal growth factor receptor, EGFR）、ROS原癌基因1受体酪氨酸激酶（ROS proto-oncogene receptor tyrosine kinase 1, ROS1）和间变性淋巴瘤激酶（anaplastic lymphoma kinase, ALK）之后的重要治疗靶点，MET异常的靶向治疗药物正日益成为关注焦点^[1]^。常见的MET基因异常包括MET 14号外显子（MET exon 14, METex14）跳跃突变、MET扩增（MET amplification）、MET蛋白过表达（MET overexpression）和MET融合（MET fusion）等，当下针对MET异常的检测手段及治疗药物正处于迅速发展阶段，因而对其在临床实践中的应用予以规范并提出相应建议实属必要^[2]^。

来自全国各个省市自治区的诸多专业人士共同构成了本共识专家组，专家组成员涵盖肺癌诊疗的多个学科。共识编写组检索了PubMed、Web of Science等英文数据库以及万方、中国知网等中文数据库中MET异常的相关文献，选取了其中证据级别较高、研究内容契合临床实践的证据，通过召开多次专家会议就MET异常检测和治疗的关键问题进行了深入讨论。结合专家丰富的临床经验，最终就8条推荐意见达成共识，共识内容及相关证据将在正文中进行详细阐述。

本共识推荐的级别为：1A级：基于高水平证据（严谨的meta分析或RCT结果），专家组有统一认识；1B级：基于高水平证据[严谨的meta分析或随机对照实验（randomized controlled trial, RCT）结果]，专家组有小争议；2A级：基于低水平证据，专家组有统一认识；2B级：基于低水平证据，专家组无统一认识，但争议不大；3级：专家组存在较大争议。

## 1 MET异常的概念和发生率

### 1.1 MET基因

MET基因位于人类7号染色体上，具有约125 kb的DNA长度，是由21个外显子与20个内含子共同组成的一种原癌基因。MET基因编码的蛋白为c-Met，主要由胞外和胞内两部分结构组成，胞外主要包含1个semaphorin（SEMA）结构域、1个plexin-semaphorin-integrin（PSI）结构域以及4个连续的immunoglobulin-plexin-transcription factors（IPT1-4）结构域；胞内主要由近膜结构域（juxtamembrane domain, JM）、酪氨酸激酶结构域（tyrosine kinase domain, TK）、C端多功能对接位点（C-terminal multifunctional docking site, MDS）三部分构成。MET蛋白亦被称为肝细胞生长因子受体（hepatocyte growth factor receptor, HGFR），属于跨膜受体酪氨酸激酶（receptor tyrosine kinase, RTK）。当HGF与c-Met结合后，会诱导c-Met发生二聚化，导致自身磷酸化并激活下游信号通路，如MAPK、PI3K/Akt和STAT3等，进而调控细胞的增殖、迁移、侵袭和血管生成。在多种癌症类型中，MET基因的异常改变可能引发癌细胞的异常增殖并显著增强癌细胞侵袭性。MET基因的异常改变主要表现为以下几个方面：（1）基因扩增：在某些癌症类型中，如肺癌、胃癌和乳腺癌等，MET基因会发生扩增，导致c-Met表达水平升高，进而激活下游信号通路，促进癌细胞的生长和侵袭；（2）基因突变：虽然MET基因的突变频率相对较低，但在某些特定类型的癌症中，如NSCLC和肾细胞癌中，已发现多种MET基因的突变形式。这些突变可能导致c-Met的持续激活，从而引发肿瘤或耐药性；（3）肝细胞生长因子（hepatocyte growth factor, HGF）过度表达：在肿瘤微环境中，肿瘤细胞和间质细胞之间的相互作用可能导致HGF的过度表达。高水平的HGF进一步激活c-Met，形成正反馈回路，加剧肿瘤细胞的恶性行为。通过深入研究MET基因的异常调控机制及其在癌症中的作用，可以为癌症的诊断、肿瘤患者的治疗和患者的预后评价提供更多可行性^[2]^。

### 1.2 METex14跳跃突变

METex14跳跃突变，是指14号外显子两侧选择性剪接位点发生突变，导致14号外显子在转录水平丢失。该突变直接导致MET蛋白JM结构域缺失，进而引发MET蛋白无法与泛素化蛋白结合，影响MET蛋白的降解，形成持续性的信号传导，促进了肿瘤细胞的增殖、迁移和侵袭^[3]^。

在NSCLC人群中，中国人群的METex14跳跃突变比例为0.9%-2.0%^[4⇓⇓-7]^，而在中国港台地区，人群METex14跳跃突变的比例分别为2.6%^[8]^和3.3%^[9]^。国际筛查数据^[10]^显示，外国人群的METex14跳跃突变比例略高于上述数据，约为2%-4%。按照不同肺癌分型分析，METex14跳跃突变在肺腺癌患者中的发生率较高，约为3%^[10]^，在肺鳞癌患者中发生率为1%-2%^[11,12]^。相关研究^[13,14]^表明，在肺肉瘤样癌患者群体中，有13%-22%会出现METex14跳跃突变。研究数据^[15]^显示，携带METex14跳跃突变的NSCLC患者同时伴有EGFR突变的比例为0.3%-10.0%，同时伴有EGFR基因扩增的比例为6.4%-28.5%。METex14跳跃突变一般多发生于老年患者^[13]^，患者中位年龄为72.5岁^[16]^。

METex14跳跃突变患者其肿瘤细胞具有高侵袭性特征，往往在抗肿瘤治疗过程中出现耐药性并且患者预后不良^[8,17]^。既往研究^[18]^显示，携带METex14跳跃突变的NSCLC患者在接受一线化疗时，其中位总生存期（overall survival, OS）仅为6.7个月，整体疗效不佳。免疫治疗在METex14跳跃突变的NSCLC患者中疗效同样也不理想，METex14跳跃突变局部晚期或转移性NSCLC患者一线应用免疫治疗的客观缓解率（objective response rate, ORR）为17%-35.7%，中位无进展生存期（progression-free survival, PFS）仅1.9-4.9个月^[19,20]^。

METex14跳跃突变的NSCLC患者通常具有较低的肿瘤突变负荷（tumor mutational burden, TMB），即便程序性细胞死亡配体1（programmed cell death ligand 1, PD-L1）阳性（≥1%）及PD-L1高表达（≥50%），但免疫单药对于这些患者的治疗效果不佳，特别是病理类型为肉瘤样癌。研究数据^[21]^已证实，即使PD-L1高表达，免疫治疗的疗效仍不理想。相对免疫治疗而言，靶向治疗的研究结果似乎能够证明其在PFS和OS上取得了一定优势。但截至目前，仍没有十分明确的一线治疗方案能够帮助PD-L1阳性及METex14跳跃突变的NSCLC患者取得显著获益，仍需要研究者不断实践和探索。

### 1.3 MET扩增

MET扩增是指MET基因在肿瘤细胞中的拷贝数显著增加。MET信号通路的激活通常是由于MET扩增导致MET蛋白过表达，从而促进肿瘤细胞生长、增殖和转移。原发MET扩增在NSCLC中发生率较低，为1%-5%^[17]^，但这一比例可能会因患者选择、检测方法和样本量等因素而有所不同。第一、二代EGFR-酪氨酸激酶抑制剂（tyrosine kinase inhibitors, TKIs）耐药导致MET基因扩增的比例为5%-22%^[22,23]^；以奥希替尼（Osimertinib）为代表的第三代EGFR-TKIs一线治疗耐药后MET基因扩增的比例为15%-20%，二线治疗耐药后为5%-50%^[24]^。MET基因扩增也是ALK-TKIs耐药的机制之一，第二、三代ALK-TKIs耐药后MET基因扩增的比例约为13%^[25]^。

### 1.4 MET蛋白过表达

MET蛋白过表达主要由以下因素引起：（1）其他致癌驱动基因的激活：MET蛋白的过表达常常作为其他致癌驱动基因（如EGFR、KRAS等）激活后的二次事件出现；（2）缺氧环境：缺氧可能通过激活缺氧诱导因子1α（hypoxia inducible factor-1α, HIF-1α）等转录因子，上调MET基因的转录水平，导致MET蛋白的过表达；（3）炎症因子：炎症因子，如肿瘤坏死因子α（tumor necrosis factor-α, TNF-α）、白介素6（interleukin-6, IL-6）等，在肿瘤微环境中大量存在，它们可以通过激活核因子κB（nuclear factor-κB, NF-κB）等信号通路，上调MET基因的表达，从而促进肿瘤的生长和转移；（4）促血管生成因子：促血管生成因子，如血管内皮生长因子（vascular endothelial growth factor, VEGF）、成纤维细胞生长因子（fibroblast growth factor, FGF）等，在肿瘤微环境中发挥重要作用，它们不仅可以促进血管生成，还可能通过激活相关的信号通路，上调MET蛋白的表达；（5）HGF：当HGF在肿瘤微环境中异常高表达时，可以持续激活MET受体，导致MET蛋白的过表达；（6）MET基因转录调控异常^[26]^。

在不同地区的NSCLC患者中，MET蛋白过表达的总体发生率呈现出较大差异，据报道^[27⇓-29]^，中国人群为17.5%-63.7%，西方人群为35%-72%，EGFR-TKIs经治的EGFR突变晚期NSCLC患者中MET蛋白过表达的发生率为30.4%-37.0%。有研究^[29,30]^显示，MET蛋白过表达在肺腺癌中的发生率甚至高达65%。

### 1.5 MET基因融合

MET基因与其他基因融合可以导致MET受体持续激活并驱动肿瘤的形成和发展。在NSCLC中，MET基因融合是一种相对罕见的分子异常，发生率在0.26%-0.5%，多为KIF5B-MET融合基因^[31]^。

### 1.6 MET活化突变

除了METex14跳跃突变外，MET激酶区、近膜区以及胞外区的错义突变同样能够导致MET受体的异常活化。在NSCLC中已发现MET胞外区E168D和N375S突变，可引起SEMA二聚化、近膜区R988C和T1010I突变以及D1020和Y1021等位点错义突变，也可参与肺癌的发生。

## 2 MET异常的检测

### 2.1 检测MET异常的必要性

#### 2.1.1 美国国立综合癌症网络（National Comprehensive Cancer Network, NCCN）、中国临床肿瘤协会（Chinese Society of Clinical Oncology, CSCO）等多个指南共识均推荐METex14跳跃突变为必检基因

国内外已有多款针对METex14跳跃突变的靶向药物获批上市，晚期NSCLC患者进行包含METex14跳跃突变的检测有助于筛选MET-TKIs靶向治疗的获益人群。2024年，包括NCCN和CSCO等机构所出版的多个国内外诊疗指南和共识中，METex14跳跃突变均已被列入了晚期NSCLC推荐检测项目，推荐级别为I或II级。除了NCCN^[32]^和CSCO^[33]^外，中华医学会肺癌临床诊疗指南2024版^[34]^、亚洲胸部肿瘤学研究小组（Asian Thoracic Oncology Research Group, ATORG）关于NSCLC MET改变的专家共识声明^[35]^、NSCLC MET临床检测中国专家共识^[36]^、IV期原发性肺癌中国治疗指南（2024版）也同样推荐^[37]^。

#### 2.1.2 NCCN、CSCO等多个指南共识推荐对于MET扩增及过表达进行检测

MET基因扩增/蛋白过表达是一种原发驱动因素，也是在EGFR-TKIs耐药进程中的关键机制之一，因此，MET基因扩增及蛋白过表达可能成为潜在的指导治疗的重要分子标志物之一。对于晚期NSCLC患者，尤其是对于EGFR-TKIs耐药的患者，其可选治疗方案有限，多项临床试验结果^[38⇓-40]^显示，携带MET基因扩增的此类患者可从MET-TKIs治疗中获益。有部分临床试验^[41,42]^也显示出MET-TKIs能够使MET蛋白过表达EGFR-TKIs耐药患者产生获益，具有值得探索的临床应用价值。基于此，目前国内外的主要诊疗指南和共识针对晚期NSCLC的扩展检测项目中已纳入MET扩增和过表达^[32⇓⇓⇓⇓-37,43]^。

**共识1：推荐所有晚期NSCLC（包括腺癌、鳞癌、肉瘤样癌等）患者在诊断时常规进行METex14跳跃突变检测（1A）；晚期初治和EGFR-TKIs耐药后NSCLC患者推荐进行MET基因扩增和蛋白过表达检测（2A）。**

### 2.2 检测MET异常的方法

#### 2.2.1 METex14跳跃突变检测方法

实时定量聚合酶链式反应（real-time quantitative polymerase chain reaction, RT-qPCR）是针对RNA的检测方法，检测针对该位点的特异性扩增产物是否存在。该方法在MET 13和15号外显子区域设计引物，其检测METex14跳跃突变的准确率高，平台可及性高，周期短，但可能存在漏检情况。例如，Y1003位点氨基酸突变（约占总体阳性的2%）或缺失等与METex14跳跃突变功能较为相似、罕见且特别的一类MET变异形式，可能会导致漏检。此外，此种方法对样本质量要求较高，需防止样本在各阶段发生RNA降解。

DNA二代测序（next-generation sequencing, NGS）以DNA作为检测的目标，运用杂交捕获法或扩增子法构建文库，以此来富集与METex14跳跃突变相关区域的片段，并进行基因序列的检测。该方法通量高，准确性较高，但检测周期往往偏长，且检出率可能会受多种因素影响，例如，引物设计、生物信息分析能力或探针覆盖等。靶向序列的建库，其覆盖范围按常规应涵盖所有的MET 13号内含子、14号外显子以及处于MET 14号内含子内且在14号外显子下游50 bp范围。另外对于NGS生物信息分析所依据的数据库要求是应包含完备的METex14跳跃突变位点信息，并且此数据库还需周期性地开展更新。

RNA NGS还可通过在RNA层面针对MET 13、15号外显子融合情况展开检测，从而判定METex14跳跃突变是否发生。此方法能够直接对METex14跳跃突变予以检测，其检测范围覆盖清晰，检测分析相对便捷。不过，该方法同样对样本的质量有着较高要求，检测所需周期较长，在可及性方面表现较弱。类似RT-qPCR，对于某些较为罕见的MET变异类型也可能存在漏检现象。

**共识2：METex14跳跃突变可使用RT-qPCR、DNA NGS或RNA NGS进行检测，不同检测方法各有其优缺点，必要时可相互验证或补充检测（1A）。**

#### 2.2.2 MET基因扩增检测方法

MET基因扩增可采用荧光原位杂交（fluorescence in situ hybridization, FISH）和DNA NGS两种检测方法。

FISH是检测MET基因扩增的金标准，可以通过两种方式定义MET扩增。一种方法依赖于确定基因拷贝数（gene copy number, GCN），即Cappuzzo标准：将MET扩增定义为每个细胞中存在5个或更多的MET拷贝数（MET GCN≥5）。该方法简单直观，适用于大多数FISH检测场景，但其局限性在于GCN无法区分真正的局部扩增与多倍体，可能导致假阳性或假阴性；第二种方法通过计算MET与7号染色体着丝粒（centromere of chromosome 7, CEP7）的比值来克服这个缺点，更准确地识别在没有染色体重复情况下的MET扩增，根据美国科罗拉多大学癌症中心（University of Colorado Cancer Center, UCCC）标准，通常使用MET/CEP7的比值≥2.0来定义扩增，并进一步将扩增细分为低（≥1.8至≤2.2）、中（>2.2至<5）、高（≥5）三个级别，有助于更精细地评估MET扩增水平^[17]^。有研究^[44]^表明MET扩增程度越高，合并其他突变的比例越低，驱动性越高，在临床试验中对MET-TKIs的治疗反应也更为显著。在该研究中，Cappuzzo标准对真正以MET基因驱动的识别能力不及MET/CEP7。

DNA NGS能够凭借测序深度以及特定位点变异频率等相关信息，来完成对MET基因拷贝数变异的计算，不过当前仍需要开展进一步的优化与验证流程。研究^[45]^表明，在检测MET扩增时，组织NGS中（当肿瘤细胞比例达到≥10%，测序深度≥500×时）的检测结果与FISH检测结果的阳性一致性约为62.5%，并且通过NGS所检测出的MET扩增情况对于临床结局难以起到有效的预测作用。

**共识3：MET基因扩增可采用FISH和DNA NGS进行检测，FISH是检测MET基因扩增的金标准，DNA NGS检测MET基因扩增尚需进一步优化和验证（2A）。**

#### 2.2.3 MET蛋白过表达检测方法

免疫组织化学法（immunohistochemistry, IHC）可作为MET蛋白过表达的检测方法。IHC的基本原理是抗原-抗体特异性结合。为了利用抗体实现对抗原定性、定位以及相对定量的目的，通常会利用化学反应让标记抗体的显色剂显色，常用的显色剂诸如酶、同位素、荧光素和金属离子等，以此明确组织细胞内的抗原。如何判断MET IHC染色强度以及区分肿瘤细胞阳性比例是此方法的核心和关键。Clinical Score标准是一种临床认可的判断标准，该标准被SAVANNAH、INSIGHT、TATTON等研究共同采用^[46]^，此标准是将染色强度与肿瘤细胞阳性比例相结合，从而完成MET IHC评分。由于目前并未明确规定MET阳性细胞比例的临界值，综合多项临床研究可见研究人员通常将50%的临界值作为常规入组标准。例如，INSIGHT研究^[47]^、TATTON研究^[48]^与SAVANNAH研究^[38]^纳入的是≥50%肿瘤细胞呈现强或中等染色的患者，相似地，SCC244-104及SCC244-108两项研究中入组的同样是≥50%肿瘤细胞强染色的患者^[20]^。

当前国内外范围内，已有多个检测MET蛋白过表达的抗体产品获准医疗器械注册或备案，这些抗体涵盖多个克隆号。不过，由于不同抗体在染色性能方面存在差别，所以至今尚未形成统一的判读标准。确定MET IHC判读标准应当将有效筛选出能够从治疗中获益的人群作为首要目标，而此方面仍然有待开展更深入的探究。

**共识4：对于EGFR-TKIs耐药的晚期NSCLC患者，推荐使用IHC进行MET蛋白过表达检测，结果判读建议参考Clinical Score标准，结合肿瘤细胞胞膜/胞质染色强度以及不同染色强度肿瘤细胞百分比进行综合判读（2B）。**

## 3 MET异常的治疗

### 3.1 METex14跳跃突变的治疗

目前多个MET-TKIs如赛沃替尼（Savolitinib）、谷美替尼（Glumetinib）、伯瑞替尼（Bozitinib）、特泊替尼（Tepotinib）、卡马替尼（Capmatinib）已在国内上市，且获得多个指南/共识推荐用于METex14跳跃突变的治疗（表1^[20,49⇓⇓⇓-53]^）。

**表1:** 治疗METex14跳跃突变的靶向药物（口服）在局部晚期或转移性NSCLC中关键临床研究数据

药物名称	治疗 线数	入组 例数	ORR （%，95%CI）	DCR （%，95%CI）	mDOR （月，95%CI）	mPFS （月，95%CI）	mOS （月，95%CI）	主要不良反应 （发生率≥20%）
赛沃替尼^[49]^	总人群	87	62 （51-72）	92 （84-97）	12.5 （8.3-15.2）	13.7 （8.5-16.6）	NE	外周水肿、恶心、低白蛋白血症、转氨酶升高、呕吐、食欲减退、乏力、低血钾、贫血、咳嗽
谷美替尼^[20,50]^	总人群	79	66 （54-76）	84 （74-91）	8.3 （6.2-NE）	8.5 （7.6-9.7）	19.4（12.1-30.1）	外周水肿、头痛、恶心、食欲减退、低白蛋白血症、谷丙转氨酶升高、呕吐
	一线	44	71 （55-83）	89 （74-91）	15.0 （6.3-NE）	11.7 （7.6-21.9）	25.4 （11.7-NA）	
	二线及以上	35	60 （42-76）	77 （60-90）	8.2 （5.1-NE）	7.6 （4.1-9.6）	16.3 （8.7-NA）	
伯瑞替尼^[51]^	总人群	52	75.0（61.1-86.0）	96.2（86.8-99.5）	16.5 （9.2-19.4）	14.3 （6.4-18.2）	20.7（16.2-29.7）	外周水肿、低白蛋白血症、低蛋白血症和贫血
	一线	35	77.1（59.9-89.6）	97.1 （85.1-99.9）	17.1 （9.2-21.8）	14.5 （6.3-20.3）	20.3 （16.2-NE）	
	二线及以上	17	70.6（44.0-89.7）	94.1（71.3-99.9）	15.3 （3.7-19.4）	7.7 （3.7-20.3）	20.7 （13.7-NE）	
特泊替尼^[52]^	总人群	313	51.4（45.9-57.1）	76.0（70.9-80.7）	18 （12.4-46.4)	11.2 （9.5-13.8）	19.6（16.2-22.9）	外周水肿、低白蛋白血症、恶心、腹泻和血肌酐水平升高
	一线	164	57.3（49.4-65.0）	78.7（71.6-84.7）	46.4 （13.8-NE）	12.6 （9.7-17.7）	21.3（14.2-25.9）	
	二线及以上	149	45.0（36.8-53.3）	73.8（66.0-80.7）	12.6 （9.5-18.5）	11.0 （8.2-13.7）	19.3（15.6-22.3）	
卡马替尼^[53]^	一线	60	68 （55.0-79.7）	98 （91.1-100.0）	16.6 （8.4-22.1）	12.5 （8.3-18.0）	21.4（15.2-30.5）	外周水肿、恶心、血肌酐升高、呕吐、呼吸困难、疲乏、食欲减退
	二线及以上	100	44 （34.1-54.3）	82 （73.1-89.0）	9.7 （5.6-13.0）	5.5 （4.2-8.1）	16.8（11.6-23.8）	

MET：间质上皮细胞转化因子；NSCLC：非小细胞肺癌；NE：未评估；ORR：客观缓解率；DCR：疾病控制率；DOR：缓解持续时间；PFS：无进展生存期；OS：总生存期。

赛沃替尼为国内自主研发的MET-TKIs，其治疗METex14跳跃突变的NSCLC的III期临床研究（NCT04923945）^[49]^报道的总体ORR为62%，中位缓解持续时间（duration of response, DOR）为12.5个月，中位PFS为13.7个月。目前赛沃替尼医保报销范围是含铂化疗后疾病进展或不耐受标准含铂化疗的、具有METex14跳跃突变的局部晚期或转移性NSCLC成人患者（二线）。

GLORY研究^[20]^是一项单臂、全球多中心、II期研究，评估了国产新型MET-TKIs谷美替尼治疗METex14跳跃突变的局部晚期或转移性NSCLC的有效性和安全性。总体受试者的ORR为66%，其中初治患者与既往经治患者的ORR分别为71%和60%；中位DOR为8.2个月，中位PFS为8.5个月，其中初治患者为11.7个月，既往经治患者为7.6个月；中位OS为19.4个月，其中初治患者为25.4个月，既往经治患者为16.3个月；基线有脑转移的13例患者中，ORR为85%（11/13），其中5例患者的脑转移病灶被选为靶病灶并在治疗后测量，该5例患者均观察到颅内部分缓解，颅内ORR为100%^[50]^。谷美替尼医保报销范围为具有METex14跳跃突变的局部晚期或转移性NSCLC（全线）。

KUNPENG研究^[51]^是一项针对局部晚期或转移性MET突变的NSCLC患者的II期开放标签、多中心、多队列研究，该研究随访2.5年，评价伯瑞替尼的有效性和安全性。该研究的总体ORR为75.0%，中位DOR为16.5个月，中位PFS为14.3个月，中位OS为20.3个月。初治患者ORR为77.1%，既往经治患者ORR为70.6%。

VISION研究^[52]^是一项针对METex14跳跃突变的局部晚期或转移性NSCLC患者的II期非随机、开放标签、多队列、多中心长期研究，旨在探索特泊替尼的疗效和安全性。根据液体活检或组织活检确定是否检测到METex14跳跃突变，结果显示，特泊替尼在总体患者人群中的ORR为51.4%，中位DOR为18个月，中位PFS为11.2个月，中位OS为19.6个月。

GEOMETRY mono-1^[53]^同样是针对METex14跳跃突变的NSCLC患者的一项II期临床研究。患者接受卡马替尼治疗，结果显示，在初治患者队列中ORR为68%，中位DOR为16.6个月，中位PFS为12.5个月，中位OS为21.4个月；既往经治患者的ORR为44%，中位DOR为9.7个月，中位PFS为5.5个月，中位OS为16.8个月。

**共识5：METex14跳跃突变的局部晚期或转移性NSCLC患者可使用谷美替尼、伯瑞替尼、特泊替尼、卡马替尼，无法耐受化疗或含铂化疗后疾病进展可使用赛沃替尼（2A）。**

### 3.2 MET原发扩增的治疗

目前有限的临床研究数据提示，MET-TKIs可能为原发性MET扩增的晚期NSCLC患者带来获益（表2^[53⇓⇓⇓-57]^）。根据MET扩增是否伴随EGFR突变，可将其分为两类：EGFR突变伴MET扩增和EGFR阴性MET扩增。

**表2:** 治疗MET原发扩增的靶向药物（口服）在局部晚期或转移性NSCLC中关键临床研究数据

药物名称	治疗线数	入组 例数	基因扩增水平	ORR (%，95%CI)	DCR (%，95%CI)	mDOR (月，95%CI)	mPFS (月，95%CI)	mOS (月，95%CI)
克唑替尼^[55]^		25	拷贝数≥6	32	52 (4个周期)		3.2 (1.9-3.7)	7.7 (4.6-15.7)
卡马替尼^[53]^	一线	15	拷贝数≥10	40 (16-68)	67 (38-88)	7.5 (2.6-14.3)	4.2（1.4-6.9)	
	二线及以上	69	拷贝数≥10	29 (19-41)	71 (59-81)	8.3 (4.2-15.4)	4.1（2.9-4.8)	
特泊替尼^[56]^	总人群	24	拷贝数≥2.5	42 (22-63)		NE (2.8-NE)	4.2（1.4-NE)	
	一线	7	拷贝数≥2.5	71 (29-96)		NE (2.8-NE)	NE（1.4-NE)	
	二线	10	拷贝数≥2.5	30 (7-65)		NE (NE-NE)	NE（1.0-NE)	
	三线	7	拷贝数≥2.5	29 (4-71)		NE (3.2-NE)	1.4（0.6-4.5)	
赛沃替尼+ 奥希替尼^[54]^	一线	21	-	90.5（69.6- 8.8）	95.2（76.2- 99.9）	18.6（NR-NE）	19.6（10.2-NE）	
谷美替尼^[57]^	总体人群	22	-	40.9（20.7-63.6）				

mDOR：中位缓解持续时间；mPFS：中位无进展生存期；mOS：中位总生存期；NR：未达到。

对于EGFR突变伴MET扩增的患者，赛沃替尼联合奥希替尼的一线治疗方案在FLOWERS研究（CTONG 2008）^[56]^中显示出显著的疗效。研究结果显示，联合治疗的ORR为90.5%，较奥希替尼单药提升29.6%，中位PFS为19.6个月，较单药延长10.3个月。

对于EGFR阴性但存在MET扩增的患者，MET-TKIs单药治疗也显示出一定疗效。AcSé研究^[55]^中，克唑替尼单药治疗原发MET扩增（GCN≥6）NSCLC的数据显示，患者用药2个周期后的ORR为16%，中位PFS为3.2个月，中位OS为7.7个月。GEOMETRY mono-1研究^[53]^探究了卡马替尼治疗MET扩增NSCLC患者的有效性。研究结果显示，卡马替尼单药治疗原发MET扩增NSCLC患者，患者MET扩增程度越高，肿瘤应答率越高。在高水平MET扩增（GCN≥10）的NSCLC患者中，初治患者ORR为40%，既往经治患者为29%。VISION研究^[56]^中，队列B探究了特泊替尼针对MET扩增晚期NSCLC患者的有效性。7例患者（29%）接受一线特泊替尼治疗，10例（42%）接受二线治疗，7例（29%）接受三线治疗。独立评审委员会（Independent Review Committee, IRC）评估的总体ORR为42%（10/24），其中，一线治疗为71%（5/7），二线治疗为30%（3/10），三线治疗为29%（2/7）。谷美替尼两项研究（NCT03457532、NCT04270591）的汇总分析结果^[57]^显示，谷美替尼单药在驱动基因阴性、MET蛋白过表达伴MET扩增亚组中，整体ORR为40.9%。

**共识6：EGFR突变伴MET扩增的晚期NSCLC患者可使用奥希替尼联合赛沃替尼进行治疗；EGFR阴性、MET扩增患者可使用卡马替尼、特泊替尼、谷美替尼或克唑替尼单药进行治疗（2B）。**

### 3.3 EGFR-TKIs耐药后MET扩增的治疗

多项临床研究数据^[38⇓⇓⇓-42,58,59]^表明，EGFR-TKIs治疗后部分患者出现MET扩增突变，化疗及免疫治疗对此类患者获益有限。针对继发性MET扩增导致的EGFR-TKIs耐药患者，EGFR-TKIs联合MET-TKIs可能是潜在的治疗策略（表3^[38⇓⇓⇓-42,58,59]^）。

**表3:** 治疗MET继发扩增的靶向药物（口服）在局部晚期或转移性NSCLC中关键临床研究数据

药物名称	分层	入组例数	ORR（%，95%CI）	mDOR（月，95%CI）	mPFS（月，95%CI）	mOS（月，95%CI）
赛沃替尼+奥希替尼^[58]^	300 mg剂量组	138	48 (39-56)	9.5 (6.9-11.2)	7.6 (5.5-9.2)	
	600 mg剂量组	36	64 (46-79)	8.0 (4.5-NR)	9.1 (5.4-12.9)	
赛沃替尼+奥希替尼^[38]^	总人群	193	32 (26-39)	8.3 (6.9-9.7)	5.3 (4.2-5.8)	
	高扩增/表达人群	108	49 (39-59)	9.3 (7.6-10.6)	7.1 (5.8-8.0)	
谷美替尼+奥希替尼^[39]^	总人群	30	60 (40.6-77.3)	5.8 (3.9-12.7)	6.9 (3.9-8.9)	16.9 (11.1-NE)
特泊替尼+吉非替尼^[40]^	总人群	12	66.7 (39.1-87.7)	19.9 (7.0-NE)	16.6 (8.3-22.1)	37.3 (21.1-52.1)
特泊替尼+奥希替尼^[41]^	总人群	98	50 (39.7-60.3)	8.5 (6.1-NE)	5.6 (4.2-8.1)	
卡马替尼+吉非替尼^[42]^	总人群		27	5.6		
REGN5093^[59]^	拷贝数≥4	20	25			

TATTON研究是一项多队列、多中心、开放标签、Ib期研究^[58]^，该研究探索了奥希替尼联合赛沃替尼双靶向治疗在第一、二代EGFR-TKIs治疗后进展且伴MET扩增的局部晚期或转移性EGFR突变阳性NSCLC患者中的效果。其中B队列患者接受奥希替尼联合赛沃替尼600 mg（n=130）或300 mg（n=8）。D队列中42例患者接受奥希替尼联合赛沃替尼300 mg治疗。B队列和D队列的ORR分别为48%和64%。

SAVANNAH II期研究^[38]^针对第三代EGFR-TKIs耐药后MET扩增和/或MET过表达的NSCLC患者。结果显示，在总人群中，赛沃替尼联合奥希替尼的双靶向治疗方案ORR为32%，中位DOR为8.3个月，中位PFS为5.3个月；MET高扩增/高过表达患者亚组（FISH10+和/或IHC90+）的获益更大，ORR达49%，中位DOR达9.3个月，中位PFS达7.1个月。

SCC244-203是一项Ib/II期研究^[39]^，评估了谷美替尼联合奥希替尼治疗第一、二、三代EGFR-TKIs耐药后MET扩增NSCLC患者的有效性和安全性。在整体人群中，ORR达到60%，疾病控制率（disease control rate, DCR）达到90%，中位PFS达6.9个月，中位OS达16.9个月。

INSIGHT研究结果^[40]^显示，与化疗相比，特泊替尼联合吉非替尼改善了EGFR-TKIs耐药后MET扩增NSCLC患者的PFS、OS、ORR和DOR：特泊替尼联合吉非替尼组和化疗组的中位PFS分别为16.6和4.2个月（HR=0.13），中位OS分别为37.3和13.1个月（HR=0.10），ORR分别为66.7%和42.9%，中位DOR分别为19.9和2.8个月。

INSIGHT 2的主要分析^[41]^显示，在伴有MET扩增且一线奥希替尼进展的EGFR突变NSCLC患者中，特泊替尼联合奥希替尼治疗的ORR为50%，中位DOR为8.5个月。不伴随其他奥希替尼耐药生物标志物的患者预后更好。

一项Ib/II期研究^[42]^评估了卡马替尼联合吉非替尼治疗EGFR-TKIs治疗失败并伴有MET扩增/过表达的NSCLC患者的有效性和安全性。II期研究的ORR为27%，中位DOR为5.6个月。在高MET扩增肿瘤的患者中，MET活性增加，MET基因GCN≥6的患者的ORR为47%。

也有研究积极探索非TKIs类药物治疗MET扩增NSCLC的效果。REGN5093是一种新型的双特异性抗体药物，同时靶向MET和EGFR。一项1/2期研究^[57]^评估了REGN5093单药治疗MET异常晚期NSCLC患者的有效性，研究发现，MET扩增患者队列的ORR为25%。

**共识7：MET继发扩增的晚期NSCLC患者可使用EGFR-TKIs联合MET-TKIs进行治疗（2A）。**

### 3.4 MET蛋白过表达的治疗

有关谷美替尼的两项研究（NCT03457532、NCT04270591）的汇总分析结果^[57]^显示，谷美替尼单药治疗驱动基因阴性MET过表达NSCLC患者的总体ORR为37.5%，中位DOR为10.3个月，中位PFS为6.9个月，中位OS为17.0个月。其中初治组ORR为41.7%，中位DOR为10.3个月，中位PFS为7.4个月，中位OS为23.5个月；既往经治患者ORR为35%，中位DOR为13.2个月，中位PFS为4.0个月，中位OS为13.9个月。MET过表达合并MET扩增亚组的ORR为40.9%，不合并MET扩增亚组的ORR为30%。

诸多研究如INSIGHT^[47]^以及TATTON^[48]^等表明，EGFR-TKIs耐药后，可能在EGFR-TKIs与MET抑制剂联合治疗方案中获益的患者其MET IHC检测显示≥50%肿瘤细胞为强阳性（3+），然而对于IHC检测≥50%的肿瘤细胞中等阳性（2+）的亚组患者，可能对联合靶向治疗的响应不足。另外，SAVANNAH研究^[45]^指出，奥希替尼耐药后，MET IHC检测≥90%肿瘤细胞为3+的患者能够在奥希替尼与赛沃替尼联合治疗中受益。

**共识8：可尝试使用谷美替尼治疗驱动基因阴性MET蛋白过表达患者，或使用EGFR-TKIs联合MET-TKIs治疗EGFR耐药后MET蛋白过表达的NSCLC患者（2B）。**

## 4 展望

### 4.1 MET扩增/过表达判读阈值

目前，不同检测方法（如FISH、NGS等）对于MET扩增/过表达的判读阈值存在显著差异。未来，随着对MET依赖性肿瘤生物学机制的深入研究，有望在这些检测方法上形成更为统一和标准化的判读标准。行业协会、研究机构以及监管机构可能会共同推动制定MET扩增/过表达的诊断共识，以确保不同实验室和研究中心之间的结果具有可比较性和可重复性。

随着NGS技术的不断发展，其在MET扩增检测中的应用将变得更加广泛和精确。新一代测序平台能够提供更高的测序深度和更广的覆盖范围，从而提高对MET扩增检测的灵敏度和特异性。液体活检技术的不断发展同样为MET扩增检测开辟了全新方向。例如，循环肿瘤DNA（circulating tumor DNA, ctDNA）检测，通过血液检测即可以非侵入性地监测肿瘤的动态变化，包括MET扩增的出现和演变。

单一的MET扩增检测可能不足以全面反映肿瘤的MET依赖性。未来，可能会结合其他生物标志物（如MET蛋白表达水平、MET突变状态等）进行联合检测，以更准确地评估肿瘤对MET靶向治疗的敏感性。这种联合检测策略有望提高治疗的精准度，减少不必要的药物暴露和副作用。

更多的临床研究将积累关于MET扩增/过表达与肿瘤预后、治疗反应之间关系的数据，这些数据将为判读阈值的制定和优化提供重要依据。通过比较不同判读阈值下的治疗效果和安全性，可以进一步细化MET扩增/过表达的分类标准，并指导临床决策。

未来的MET扩增/过表达判读阈值将更加注重个体化和精准化。通过结合患者的具体病情、肿瘤分子特征以及治疗效果等因素，为患者精准定制适配度最高的治疗策略。这对于提升肿瘤治疗的综合效益以及患者的生存率具有关键意义，并推动肿瘤治疗向更加个体化和精准化的方向发展。

### 4.2 原发扩增治疗

目前如卡马替尼和特泊替尼等MET-TKIs已显示出在MET扩增NSCLC患者中的有效性。未来可以进一步优化这些药物的给药方式和剂量，以提高疗效并减少副作用。持续探索和开发新的、更为高效的MET抑制剂，针对MET扩增的不同水平和表现形式，为患者提供更具有个性化和精准性的治疗方案。

联合治疗策略：MET扩增常与EGFR、ALK等其他致癌基因变异共存，联合使用MET-TKIs和相应的靶向药物可能实现更好的治疗效果。

MET扩增的水平可能影响其作为致癌驱动基因的作用。对于MET扩增水平较高的患者，MET抑制剂可能表现出更显著的治疗效果。因此，基于MET扩增水平的患者分群和治疗选择将更加精准。在治疗过程中应定期监测患者的MET扩增状态、治疗反应及可能出现的耐药突变，及时调整治疗方案。

### 4.3 MET蛋白过表达治疗

MET蛋白过表达和EGFR突变在NSCLC中可共存，这为双靶点治疗提供了强有力的理论依据。经EGFR-TKIs治疗的具有EGFR突变且伴随MET过表达的NSCLC患者，Teliso-V显示出令人鼓舞的抗肿瘤活性和可接受的毒性。Teliso-V是首个针对MET的抗体药物偶联物（antibody-drug conjugate, ADC），有两项研究^[60,61]^分别评估了Teliso-V联合奥希替尼或厄洛替尼治疗经治的MET蛋白过表达NSCLC的有效性和安全性。初步结果显示，Teliso-V联合奥希替尼的ORR为58%；Teliso-V联合厄洛替尼的研究纳入MET蛋白过表达（H-score≥150）超过50%且接受过三线及以上的EGFR-TKIs治疗的患者，总体ORR为30.6%，MET高度过表达（H-score≥225）患者亚组ORR为52.6%。其中合并MET扩增患者治疗反应良好，ORR为62.5%。未来需要更大规模的II和III期临床试验来进一步验证Teliso-V联合MET-TKIs在特定患者群体中的疗效和安全性。鉴于MET在EGFR-TKIs耐药中的潜在作用，未来可以探索Teliso-V与其他EGFR-TKIs（如奥希替尼）的联合使用，以克服EGFR-TKIs的获得性耐药。

对于晚期NSCLC且EGFR突变伴有原发MET蛋白过表达的患者，单克隆抗体Emibetuzumab与厄洛替尼联用显示出显著的临床获益。另有研究^[62]^发现，在未经治疗的EGFR突变合并原发MET过表达的肺腺癌中，存在EGFR L858R突变的患者使用EGFR-TKIs单药治疗与不存在L858R突变的患者相比预后较差，原发MET过表达合并EGFR L858R突变的患者是否可以从EGFR-TKIs联合MET-TKIs治疗中获益值得进一步探索。

在NSCLC驱动基因阴性伴MET蛋白过表达的患者治疗中，谷美替尼显示出与标准化疗、其他MET抑制剂如BPI-9016M^[63]^和Tivantinib^[64]^以及抗体Onartuzumab^[65]^相比更佳的疗效，但其在该患者人群中的治疗获益仍需进一步验证。一项开放标签、多中心、随机、III期研究（CTR20230451）评估谷美替尼对比多西他赛在免疫治疗和铂类化疗后进展的驱动基因阴性且伴有MET过表达的局部晚期或转移性NSCLC患者中的疗效，该研究目前正在进行中。

另外还需深入研究MET蛋白过表达和其他相关生物标志物的预测价值，以优化患者选择和个性化治疗策略。基于生物标志物（如MET H-score、EGFR突变状态等）的精准治疗策略，将有助于筛选出最可能从MET过表达治疗中获益的患者。

### 4.4 MET融合治疗

针对MET融合的靶向治疗药物目前主要是MET-TKIs。近期一项研究^[66]^不仅确认了已知的MET融合伴侣，还首次发现了EPHB4、THAP5、TNPO3和DST作为MET的新型融合伴侣。值得注意的是，携带新型EPHB4-MET融合基因的患者，MET-IHC、MET-FISH和METex14跳跃突变检测均为阴性，且患者接受MET-TKIs治疗后表现出持续抗肿瘤应答，证明EPHB4-MET融合可以作为潜在治疗靶点。研究中12例患者接受MET-TKIs治疗，表现出了显著的治疗应答差异：6例患者实现了部分缓解，2例患者病情稳定，4例患者疾病进展，提示不同MET融合类型的患者对MET-TKIs的响应存在差异。EPHB4-MET融合被证实在某些情况下能够介导对EGFR-TKIs和ALK-TKIs的抵抗，而MET-DST融合则可能与METex14跳跃突变同时存在，影响治疗效果。

在治疗过程中，患者可能会出现对MET-TKIs的继发性耐药。患者可能在获得性耐药后携带MET D1228H/N或D1246N等突变，这些突变影响了抑制剂的治疗效果。为了应对耐药性和提高治疗效果，需要不断探索新的MET-TKIs和联合治疗方案。例如，Tivantinib在肿瘤类器官（patient-derived organoid, PDO）模型中表现出对携带MET D1228N突变的患者具有较好的抑制效果。此外，通过深入了解MET融合的分子机制，可以开发出针对特定融合形式的特异性抑制剂，从而提高治疗效果。

### 4.5 MET异常的其他类型

除上述4种主要类型外，MET基因异常还包括MET激酶结构域（MET tyrosine kinase domain, MET TKD）突变和MET激酶结构域复制（MET kinase domain duplication, MET KDD）等。在2022年美国临床肿瘤学会（American Society of Clinical Oncology, ASCO）大会上，一项关于MET TKD突变的研究结果显示：无并发METex14跳跃突变的MET TKD突变在NSCLC中的检出率为0.6%，而其中只有21.4%具有明确的致病意义，可见这类突变在NSCLC中非常罕见。值得一提的是，MET TKD突变在临床中往往和MET-TKIs类药物的耐药相关。目前，虽然已有多款MET靶向药物获批上市，用于METex14跳跃突变NSCLC的治疗，但对于耐药后出现MET TKD突变的患者，尚缺乏标准的临床治疗方案。KDD是基因内部分复制的一种，为肿瘤细胞受体酪氨酸激酶的激活提供了一种新的机制，如EGFR KDD和MET KDD等。KDD在肿瘤中总体发生率为0.62%。2019年美国癌症研究协会（American Association for Cancer Research, AACR）大会^[67]^上公布了一项纳入11例MET KDD患者的研究，其中肺癌6例（0.09%）、其他肿瘤5例（0.28%），研究发现肺癌患者对MET-TKIs有部分反应。

### 4.6 MET异常患者靶向进展后的后续治疗

目前尚未明确MET-TKIs的耐药机制。有研究^[68⇓-70]^认为，MET TKD突变会削弱药物与MET TKD之间的化学键，从而产生MET-TKIs耐药性，也称作靶点依赖性耐药（on-target resistance），也存在因ERBB家族基因扩增导致旁路和/或下游信号激活造成脱靶耐药（off-target resistance），或有患者同时表现靶点依赖性耐药和脱靶耐药两种机制。对于靶点依赖性耐药的患者，在I和II型MET-TKIs之间切换或可对患者产生部分响应。一项临床研究^[70]^显示，卡马替尼对克唑替尼治疗后MET突变NSCLC患者有一定的疗效，该研究纳入20例患者，包括15例METex14跳跃突变患者、5例MET扩增患者，其中有2例对卡马替尼达到客观缓解，14例患者病情稳定，DCR为80%。此外，CHRYSALIS I期研究评估了埃万妥单抗（Amivantamab）对METex14跳跃突变患者治疗的安全性和有效性，初步数据显示，该药对既往治疗无效及接受过治疗（包括接受过 MET-TKIs 治疗）的患者具有抗肿瘤活性^[71,72]^。而对于脱靶突变，将MET-TKIs与另一种靶向疗法结合可能是一种合适的方法，但仍需要进一步研究。

中国的研究者^[73]^也对部分中国医师针对MET异常NSCLC患者的临床诊疗现状进行了调研。结合调研结果，可获得中国医师对于经MET-TKIs治疗后进展或耐药的METex14跳跃突变的NSCLC患者使用何种后续治疗方案的选择情况。其中近半数医师（45.7%）选择化疗联合抗血管生成治疗，20.7%的医师选择MET-TKIs联合化疗，而12.9%的医师选择MET-TKIs联合抗血管生成治疗。

### 4.7 早期MET异常NSCLC的治疗

目前，在MET-TKIs应用于MET异常的早期NSCLC患者围手术期方面，仍缺少高证据级别的研究予以支撑。一项II期临床试验ARM研究（NCT03088930），主要针对可实施手术切除且ALK、ROS1或MET基因阳性的NSCLC患者，用以评估克唑替尼在新辅助治疗中的效果。另有一项II期临床试验Geometry-N研究（NCT04926831），聚焦于卡马替尼应用于METex14跳跃突变或MET扩增的NSCLC患者的围手术期的情况。截至目前，这两项研究的相关数据均尚未公开发表。

有个案报道^[74⇓⇓⇓-78]^显示，MET-TKIs在MET异常的早期NSCLC中取得了一定的疗效。例如，Rotow等^[74]^报告了1例右上肺肿物直径达15 cm的亚裔女性患者，该患者同时伴有纵隔淋巴结肿大的症状，且存在METex14跳跃突变及MET扩增。患者使用克唑替尼治疗8个月后接受手术治疗，经病理检查证实患者达到完全缓解，并且手术后继续使用克唑替尼作为辅助治疗方案。整个药物治疗过程中患者的耐受性表现良好，随访至术后6个月患者预后良好，未出现复发等情况。此外，仍有多篇关于MET-TKIs应用于新辅助治疗的病例报告^[75⇓⇓-78]^，在这些案例中，患者在接受了术前4-10周的赛沃替尼治疗后，成功实现了缩瘤降期的效果，从而得以进行根治性手术切除。针对术后的辅助治疗，这些案例分别采用了不同的策略，Zhang等^[75]^和Deng等^[76]^报道了持续使用赛沃替尼对患者辅助治疗的病例，Tian等^[77]^报道了在降期后依据患者术后分期决定不实施辅助治疗而仅作持续观察的病例，还有Fu等^[78]^报道了在术后对患者采用标准的培美曲塞加卡铂辅助化疗的病例。

基于已有的个案报道，对于年龄较大、拒绝化疗或存在化疗禁忌证的患者，可考虑进行MET-TKIs的探索性应用。同时，对于早期NSCLC且伴有MET突变的患者，期待开展高质量的前瞻性多中心临床试验项目，以此来深度探究MET-TKIs在围手术期治疗进程中的安全性以及有效性。

总之，MET异常在NSCLC人群中基数巨大，METex14跳跃突变已经通过临床研究并获得了高证据级别的适应证批准，其他MET异常，尤其是MET扩增和MET过表达人群数量更加庞大，更需要加速通过高证据级别的研究获得临床适应证的批准，最终给患者带来更便利的应用指导。

## References

[1] Friedlaender A, Perol M, Banna GL, et al. Oncogenic alterations in advanced NSCLC: a molecular super-highway. Biomark Res, 2024, 12(1): 24. doi: 10.1186/s40364-024-00566-0 PMC10863183 38347643

[2] Friedlaender A, Drilon A, Banna GL, et al. The METeoric rise of MET in lung cancer. Cancer, 2020, 126(22): 4826-4837. doi: 10.1002/cncr.33159 32888330 PMC9066984

[3] Santarpia M, Massafra M, Gebbia V, et al. A narrative review of MET inhibitors in non-small cell lung cancer with MET exon 14 skipping mutations. Transl Lung Cancer Res, 2021, 10(3): 1536-1556. doi: 10.21037/tlcr-20-1113 33889528 PMC8044480

[4] Liu SY, Gou LY, Li AN, et al. The unique characteristics of MET exon 14 mutation in Chinese patients with NSCLC. J Thorac Oncol, 2016, 11(9): 1503-1510. doi: 10.1016/j.jtho.2016.05.016 27257131

[5] Qiu T, Li W, Zhang T, et al. Distinct MET protein localization associated with MET exon 14 mutation types in patients with non-small-cell lung cancer. Clin Lung Cancer, 2018, 19(4): e391-e398. doi: 10.1016/j.cllc.2017.12.006 29338938

[6] Xu Z, Li H, Dong Y, et al. Incidence and PD-L1 expression of MET 14 skipping in Chinese population: A non-selective NSCLC cohort study using RNA-based sequencing. Onco Targets Ther, 2020, 13: 6245-6253. doi: 10.2147/OTT.S241231 32669854 PMC7335768

[7] Zheng D, Wang R, Ye T, et al. MET exon 14 skipping defines a unique molecular class of non-small cell lung cancer. Oncotarget, 2016, 7(27): 41691-41702. doi: 10.18632/oncotarget.9541 27223439 PMC5173088

[8] Tong JH, Yeung SF, Chan AW, et al. MET amplification and exon 14 splice site mutation define unique molecular subgroups of non-small cell lung carcinoma with poor prognosis. Clin Cancer Res, 2016, 22(12): 3048-3056. doi: 10.1158/1078-0432.CCR-15-2061 26847053

[9] Gow CH, Hsieh MS, Wu SG, et al. A comprehensive analysis of clinical outcomes in lung cancer patients harboring a MET exon 14 skipping mutation compared to other driver mutations in an East Asian population. Lung Cancer, 2017, 103: 82-89. doi: 10.1016/j.lungcan.2016.12.001 28024701

[10] Liang H, Wang M. MET oncogene in non-small cell lung cancer: Mechanism of MET dysregulation and agents targeting the HGF/c-Met axis. Onco Targets Ther, 2020, 13: 2491-2510. doi: 10.2147/OTT.S231257 32273721 PMC7104217

[11] Schrock AB, Frampton GM, Suh J, et al. Characterization of 298 patients with lung cancer harboring MET exon 14 skipping alterations. J Thorac Oncol, 2016, 11(9): 1493-1502. doi: 10.1016/j.jtho.2016.06.004 27343443

[12] Lam VK, Tran HT, Banks KC, et al. Targeted tissue and cell-free tumor DNA sequencing of advanced lung squamous-cell carcinoma reveals clinically significant prevalence of actionable alterations. Clin Lung Cancer, 2019, 20(1): 30-36.e3. doi: 10.1016/j.cllc.2018.08.020 30279110

[13] Vuong HG, Ho A, Altibi A, et al. Clinicopathological implications of MET exon 14 mutations in non-small cell lung cancer - A systematic review and meta-analysis. Lung Cancer, 2018, 123: 76-82. doi: 10.1016/j.lungcan.2018.07.006 30089599

[14] Liu X, Jia Y, Stoopler MB, et al. Next-generation sequencing of pulmonary sarcomatoid carcinoma reveals high frequency of actionable MET gene mutations. J Clin Oncol, 2016, 34(8): 794-802. doi: 10.1200/JCO.2015.62.0674 26215952

[15] Fujino T, Suda K, Mitsudomi T. Lung cancer with MET exon 14 skipping mutation: Genetic feature, current treatments, and future challenges. Lung Cancer (Auckl), 2021, 12: 35-50. doi: 10.2147/LCTT.S269307 34295201 PMC8290191

[16] Awad MM, Oxnard GR, Jackman DM, et al. MET exon 14 mutations in non-small-cell lung cancer are associated with advanced age and stage-dependent MET genomic amplification and c-Met overexpression. J Clin Oncol, 2016, 34(7): 721-730. doi: 10.1200/JCO.2015.63.4600 26729443

[17] Guo R, Luo J, Chang J, et al. MET-dependent solid tumours - molecular diagnosis and targeted therapy. Nat Rev Clin Oncol, 2020, 17(9): 569-587. doi: 10.1038/s41571-020-0377-z 32514147 PMC7478851

[18] Drilon A, Clark JW, Weiss J, et al. Antitumor activity of crizotinib in lung cancers harboring a MET exon 14 alteration. Nat Med, 2020, 26(1): 47-51. doi: 10.1038/s41591-019-0716-8 31932802 PMC8500676

[19] Paik PK, Felip E, Veillon R, et al. Tepotinib in non-small-cell lung cancer with MET exon 14 skipping mutations. N Engl J Med, 2020, 383(10): 931-943. doi: 10.1056/NEJMoa2004407 32469185 PMC8422679

[20] Yu Y, Zhou J, Li X, et al. Gumarontinib in patients with non-small-cell lung cancer harbouring MET exon 14 skipping mutations: a multicentre, single-arm, open-label, phase 1b/2 trial. EClinicalMedicine, 2023, 59: 101952. doi: 10.1016/j.eclinm.2023.101952 37096188 PMC10121392

[21] Lung Cancer Specialty Committee of Chinese Elderly Health Care Association. Expert consensus on targeted therapy of NSCLC with MET exon 14 skipping mutation. Zhongguo Feiai Zazhi, 2023, 26(6): 416-428. 37488079 10.3779/j.issn.1009-3419.2023.102.19 PMC10365964

[22] Westover D, Zugazagoitia J, Cho BC, et al. Mechanisms of acquired resistance to first- and second-generation EGFR tyrosine kinase inhibitors. Ann Oncol, 2018, 29(suppl_1): i10-i19. doi: 10.1093/annonc/mdx703 PMC6454547 29462254

[23] Matikas A, Mistriotis D, Georgoulias V, et al. Current and future approaches in the management of non-small-cell lung cancer patients with resistance to EGFR TKIs. Clin Lung Cancer, 2015, 16(4): 252-261. doi: 10.1016/j.cllc.2014.12.013 25700775

[24] Leonetti A, Sharma S, Minari R, et al. Resistance mechanisms to osimertinib in EGFR-mutated non-small cell lung cancer. Br J Cancer, 2019, 121(9): 725-737. doi: 10.1038/s41416-019-0573-8 31564718 PMC6889286

[25] Dagogo-Jack I, Yoda S, Lennerz JK, et al. MET alterations are a recurring and actionable resistance mechanism in ALK-positive lung cancer. Clin Cancer Res, 2020, 26(11): 2535-2545. doi: 10.1158/1078-0432.CCR-19-3906 32086345 PMC7269872

[26] Yu XQ, Xu YJ, Fan Y. Progress of c-MET signaling pathway and TKIs in non-small cell lung cancer. Zhongguo Feiai Zazhi, 2017, 20(4): 287-292. 28442019 10.3779/j.issn.1009-3419.2017.04.10 PMC5999681

[27] Park S, Choi YL, Sung CO, et al. High MET copy number and MET overexpression: poor outcome in non-small cell lung cancer patients. Histol Histopathol, 2012, 27(2): 197-207. doi: 10.14670/HH-27.197 22207554

[28] Lv H, Shan B, Tian Z, et al. Soluble c-Met is a reliable and sensitive marker to detect c-Met expression level in lung cancer. Biomed Res Int, 2015, 2015: 626578. doi: 10.1155/2015/626578 25834821 PMC4365312

[29] Li XF, Chen ZW, Xi YF, et al. Correlation between expression of C-met and epidermal growth factor receptor-tyrosine kinase inhibitor resistance in lung adenocarcinoma. Zhongliu Yanjiu Yu Linchuang, 2018, 30(1): 1-6.

[30] Watermann I, Schmitt B, Stellmacher F, et al. Improved diagnostics targeting c-MET in non-small cell lung cancer: expression, amplification and activation?. Diagn Pathol, 2015, 10: 130. doi: 10.1186/s13000-015-0362-5 26215852 PMC4517562

[31] Zhuo M, Liang Z, Yi Y, et al. Analysis of MET kinase domain rearrangement in NSCLC. Lung Cancer, 2020, 145: 140-143. doi: 10.1016/j.lungcan.2020.04.040 32447117

[32] NCCN guidelines. Non-small cell lung cancer version 8. 2024. https://www.nccn.org/guidelines/guidelines-detail?category=1&id=1450 https://www.nccn.org/guidelines/guidelines-detail?category=1&id=1450. Accessed Nov 12, 2024.

[33] Working Committee of the Chinese Society of Clinical Oncology Guidelines. Chinese Society of Clinical Oncology (CSCO) guidelines for the diagnosis and treatment of non small cell lung cancer 2024. 1^st^ ed.ed. Beijing: People's Health Publishing House, 2024.

[34] Chinese Medical Association Chinese Society of Oncology. Chinese Medical Association guideline for clinical diagnosis and treatment of lung cancer for patients (2024 edition). Zhonghua Zhongliu Zazhi, 2024, 46(9): 805-843. 39045625 10.3760/cma.j.cn112152-20240510-00189

[35] Ahn MJ, Mendoza M, Pavlakis N, et al. Asian Thoracic Oncology Research Group (ATORG) expert consensus statement on MET alterations in NSCLC: Diagnostic and therapeutic considerations. Clin Lung Cancer, 2022, 23(8): 670-685. doi: 10.1016/j.cllc.2022.07.012 36151006

[36] Chinese Society of Pathology, Pathology Quality Control Center, Lung Cancer Group of Chinese Medical Association Chinese Society of Oncology, et al. Chinese expert consensus on clinical practice of MET detection in non-small cell lung cancer. Zhonghua Binglixue Zazhi, 2022, 51(11): 1094-1103. 10.3760/cma.j.cn112151-20220606-00491 36323537

[37] Medical Oncology Branch of China International Exchange and Promotive Association for Medical and Health Care, Chinese Association for Clinical Oncologists. China clinical practice guideline for stage IV primary lung cancer (2024 edition). Zhonghua Zhongliu Zazhi, 2024, 46(7): 595-636. 39034799 10.3760/cma.j.cn112152-20240311-00104

[38] Ahn MJ, De Marinis F, Bonanno L, et al. EP08.02-140 MET biomarker-based preliminary efficacy analysis in SAVANNAH: savolitinib+osimertinib in EGFRm NSCLC post-osimertinib. J Thorac Oncol, 2022, 17(9 Supplement): S469-S470. doi: 10.1016/j.jtho.2022.07.823

[39] Yu Y, Yang N, Zhang Y, et al. SCC244 plus osimertinib in patients with stage IIIB/IIIC or IV, EGFR TKI resistant EGFR-mutant NSCLC harboring MET amplification. ESMO ASIA 2022: 305 MO. doi: 10.1016/j.annonc.2022.10.334

[40] Ohara R, Liam CK, Ahmad AR, et al. PPD02.02 Tepotinib+gefitinib in patients with EGFR-mutant NSCLC with MET amplification (METamp): Final analysis of INSIGHT. J Thorac Oncol, 2023, 18(3 Supplement): e6-e7. doi: 10.1016/j.jtho.2022.09.020

[41] Kim TM, Guarneri V, Jye VP, et al. Tepotinib+osimertinib in EGFR-mutant NSCLC with MET amplification following 1L osimertinib: INSIGHT 2 primary analysis. WCLC 2023: OA21.05. doi: 10.1016/j.jtho.2023.09.106

[42] Wu YL, Zhang L, Kim DW, et al. Phase Ib/II study of capmatinib (INC280) plus gefitinib after failure of epidermal growth factor receptor (EGFR) inhibitor therapy in patients with EGFR-mutated, MET factor-dysregulated non-small-cell lung cancer. J Clin Oncol, 2018, 36(31): 3101-3109. doi: 10.1200/JCO.2018.77.7326 30156984

[43] Molecular Pathology Collaboration Group of Tumor, Pathology Committee of Chinese Anti-Cancer Association. Chinese expert consensus on MET immunohistochemistry detection and interpretation standards for non-small cell lung cancer (2023 version). Zhonghua Binglixue Zazhi, 2023, 52(11): 1090-1097. 10.3760/cma.j.cn112151-20230626-00423 37899313

[44] Noonan SA, Berry L, Lu X, et al. Identifying the appropriate FISH criteria for defining MET copy number-driven lung adenocarcinoma through oncogene overlap analysis. J Thorac Oncol, 2016, 11(8): 1293-1304. doi: 10.1016/j.jtho.2016.04.033 27262212 PMC5404374

[45] Peng LX, Jie GL, Li AN, et al. MET amplification identified by next-generation sequencing and its clinical relevance for MET inhibitors. Exp Hematol Oncol, 2021, 10(1): 52. doi: 10.1186/s40164-021-00245-y PMC8579577 34758872

[46] Koeppen H, Yu W, Zha J, et al. Biomarker analyses from a placebo-controlled phase II study evaluating erlotinib±onartuzumab in advanced non-small cell lung cancer: MET expression levels are predictive of patient benefit. Clin Cancer Res, 2014, 20(17): 4488-4498. doi: 10.1158/1078-0432.CCR-13-1836 24687921 PMC4504679

[47] Wu YL, Cheng Y, Zhou J, et al. Tepotinib plus gefitinib in patients with EGFR-mutant non-small-cell lung cancer with MET overexpression or MET amplification and acquired resistance to previous EGFR inhibitor (INSIGHT study): an open-label, phase 1b/2, multicentre, randomised trial. Lancet Respir Med, 2020, 8(11): 1132-1143. doi: 10.1016/S2213-2600(20)30154-5 32479794

[48] Hartmaier RJ, Markovets AA, Ahn MJ, et al. Osimertinib+savolitinib to overcome acquired MET-mediated resistance in epidermal growth factor receptor-mutated, MET-amplified non-small cell lung cancer: TATTON. Cancer Discov, 2023, 13(1): 98-113. doi: 10.1158/2159-8290.CD-22-0586 36264123 PMC9827108

[49] Yu Y, Guo Q, Zhang Y, et al. Savolitinib in patients in China with locally advanced or metastatic treatment-naive non-small-cell lung cancer harbouring MET exon 14 skipping mutations: results from a single-arm, multicohort, multicentre, open-label, phase 3b confirmatory study. Lancet Respir Med, 2024, 12(12): 958-966. doi: 10.1016/S2213-2600(24)00211-X 39270695

[50] Yu Y, Zhou J, Li X, et al. Efficacy and safety outcomes of phase II study of SCC244 in NSCLC patients harboring MET exon 14 skipping (METex14) mutations (GLORY study): long-term follow-up analysis. Ann Oncol, 2024, 35(suppl_4): S1648. doi: 10.1016/j.annonc.2024.10.684

[51] Yang JJ, Zhang Y, Wu L, et al. Efficacy and safety of vebreltinib in patients with advanced NSCLC harboring MET exon 14-skipping: Results of 2.5-year follow-up in KUNPENG. J Clin Oncol, 2024, 42(16_suppl): 8557. doi: 10.1200/JCO.2024.42.16_suppl.8557

[52] Mazieres J, Paik PK, Garassino MC, et al. Tepotinib treatment in patients with MET exon 14-skipping non-small cell lung cancer: Long-term follow-up of the VISION phase 2 nonrandomized clinical trial. JAMA Oncol, 2023, 9(9): 1260-1266. doi: 10.1001/jamaoncol.2023.1962 37270698 PMC10240398

[53] Wolf J, Hochmair M, Han JY, et al. Capmatinib in MET exon 14-mutated non-small-cell lung cancer: final results from the open-label, phase 2 GEOMETRY mono-1 trial. Lancet Oncol, 2024, 25(10): 1357-1370. doi: 10.1016/S1470-2045(24)00441-8 39362249

[54] Moro-Sibilot D, Cozic N, Pérol M, et al. Crizotinib in c-MET- or ROS1-positive NSCLC: results of the AcSé phase II trial. Ann Oncol, 2019, 30(12): 1985-1991. doi: 10.1093/annonc/mdz407 31584608

[55] Yang J, Li A, Feng WN, et al. Osimertinib with or without Savolitinib as 1L in de novo MET aberrant, EGFRm advanced NSCLC (CTONG 2008, FLOWERS): A phase II trial. 2024 WCLC: PL04.10.

[56] Le X, Paz-Ares LG, Meerbeeck JV, et al. Tepotinib in patients (pts) with advanced non-small cell lung cancer (NSCLC) with MET amplification ( MET amp). J Clin Oncol, 2021, 39(15_suppl): 9021. doi: 10.1200/JCO.2021.39.15_suppl.9021

[57] Sequist LV, Han JY, Ahn MJ, et al. Osimertinib plus savolitinib in patients with EGFR mutation-positive, MET-amplified, non-small-cell lung cancer after progression on EGFR tyrosine kinase inhibitors: interim results from a multicentre, open-label, phase 1b study. Lancet Oncol, 2020, 21(3): 373-386. doi: 10.1016/S1470-2045(19)30785-5 32027846

[58] Yu Y, Dong W, Shi Y, et al. A pooled analysis of clinical outcome in driver-gene negative non-small cell lung cancer patients with MET overexpression treated with gumarontinib. Ther Adv Med Oncol, 2024, 16: 17588359241264730. doi: 10.1177/17588359241264730 39091606 PMC11292687

[59] Cho BC, Ahn M, Kim TM, et al. Early safety, tolerability, and efficacy of REGN5093 in patients (pts) with MET-altered advanced non-small cell lung cancer (aNSCLC) from a first in human (FIH) study. Ann Oncol, 2022, 33(suppl_7): S1085. doi: 10.1016/j.annonc.2022.07.1296

[60] Camidge DR, Barlesi F, Goldman JW, et al. Phase Ib study of telisotuzumab vedotin in combination with erlotinib in patients with c-Met protein-expressing non-small-cell lung cancer. J Clin Oncol, 2023, 41(5): 1105-1115. doi: 10.1200/JCO.22.00739 36288547 PMC9928626

[61] Goldman JW, Horinouchi H, Cho BC, et al. Phase 1/1b study of telisotuzumab vedotin (Teliso-V)+osimertinib (Osi), after failure on prior Osi, in patients with advanced, c-Met overexpressing, EGFR-mutated non-small cell lung cancer (NSCLC). J Clin Oncol, 2022, 40(16_suppl): 9013. doi: 10.1200/JCO.2022.40.16_suppl.9013

[62] Wang N, Zhang Y, Wu J, et al. MET overexpression correlated with prognosis of EGFR-mutant treatment-naive advanced lung adenocarcinoma: a real-world retrospective study. Clin Transl Oncol, 2024, 26(7): 1696-1707. doi: 10.1007/s12094-024-03391-x 38430418

[63] Hu X, Cui X, Wang Z, et al. Safety, efficacy and pharmacokinetics of BPI-9016M in c-MET overexpression or MET exon 14 skipping mutation patients with locally advanced or metastatic non-small-cell lung cancer: a phase Ib study. BMC Cancer, 2023, 23(1): 331. doi: 10.1186/s12885-022-10500-y PMC10088252 37041472

[64] Scagliotti G, von Pawel J, Novello S, et al. Phase III multinational, randomized, double-blind, placebo-controlled study of tivantinib (ARQ 197) plus erlotinib versus erlotinib alone in previously treated patients with locally advanced or metastatic nonsquamous non-small-cell lung cancer. J Clin Oncol, 2015, 33(24): 2667-2674. doi: 10.1200/JCO.2014.60.7317 26169611

[65] Spigel DR, Edelman MJ, O'Byrne K, et al. Results from the phase III randomized trial of onartuzumab plus erlotinib versus erlotinib in previously treated stage IIIB or IV non-small-cell lung cancer: METLung. J Clin Oncol, 2017, 35(4): 412-420. doi: 10.1200/JCO.2016.69.2160 27937096

[66] Kang J, Deng QM, Feng W, et al. Response and acquired resistance to MET inhibitors in de novo MET fusion-positive advanced non-small cell lung cancer. Lung Cancer, 2023, 178: 66-74. doi: 10.1016/j.lungcan.2023.01.017 36806896

[67] Xu CW, Wang WX, Wang XJ, et al. Abstract 1597: Real-world large-scale study kinase domain duplications across diverse tumor types in Chinese populations. Cancer Res, 2019, 79(13_Supplement): 1597. doi: 10.1158/1538-7445.AM2019-1597

[68] Bahcall M, Sim T, Paweletz CP, et al. Acquired METD1228V mutation and resistance to MET inhibition in lung cancer. Cancer Discov, 2016, 6(12): 1334-1341. doi: 10.1158/2159-8290.CD-16-0686 27694386 PMC5140694

[69] Heist RS, Sequist LV, Borger D, et al. Acquired resistance to crizotinib in NSCLC with MET exon 14 skipping. J Thorac Oncol, 2016, 11(8): 1242-1245. doi: 10.1016/j.jtho.2016.06.013 27343442

[70] Recondo G, Bahcall M, Spurr LF, et al. Molecular mechanisms of acquired resistance to MET tyrosine kinase inhibitors in patients with MET exon 14-mutant NSCLC. Clin Cancer Res, 2020, 26(11): 2615-2625. doi: 10.1158/1078-0432.CCR-19-3608 32034073

[71] Dagogo-Jack I, Moonsamy P, Gainor JF, et al. A phase 2 study of capmatinib in patients with MET-altered lung cancer previously treated with a MET inhibitor. J Thorac Oncol, 2021, 16(5): 850-859. doi: 10.1016/j.jtho.2021.01.1605 33545388 PMC8922989

[72] Krebs M, Spira A, Cho B, et al. Amivantamab in patients with NSCLC with MET exon 14 skipping mutation: Updated results from the CHRYSALIS study. J Clin Oncol, 2022, 40(16_suppl): 9008. doi: 10.1200/JCO.2022.40.16_suppl.9008

[73] Gan B, Liu SY, Yan HH, et al. Current situation investigation of clinical diagnosis and treatment of non-small cell lung cancer with MET alterations in China. Xunzheng Yixue, 2024, 24(2): 97-106.

[74] Rotow JK, Woodard GA, Urisman A, et al. Pathologic complete response to neoadjuvant crizotinib in a lung adenocarcinoma patient with a MET exon 14 skipping mutation. Clin Lung Cancer, 2019, 20(2): e137-e141. doi: 10.1016/j.cllc.2018.11.003 30553716

[75] Zhang Y, Zhang H, Wang H, et al. Use of savolitinib as neoadjuvant therapy for non-small cell lung cancer patient with MET exon 14 skipping alterations: A case report. Front Oncol, 2022, 12: 968030. doi: 10.3389/fonc.2022.968030 36176406 PMC9514319

[76] Deng HY, Qiu XM, Zhu DX, et al. The safety and feasibility of preoperative induction therapy of savolitinib in non-small cell lung cancer patients with MET exon 14 skipping mutation. J Cancer Res Clin Oncol, 2023, 149(8): 4623-4628. doi: 10.1007/s00432-022-04370-x 36171456 PMC11798222

[77] Tian J, Lin Z, Chen Y, et al. Dramatic response to neoadjuvant savolitinib in marginally resectable lung adenocarcinoma with MET exon 14 skipping mutation: A case report and literature review. Front Oncol, 2022, 12: 1006634. doi: 10.3389/fonc.2022.1006634 36387081 PMC9646987

[78] Fu M, Feng CM, Xia DQ, et al. Neoadjuvant savolitinib targeted therapy stage IIIA-N2 primary lung adenocarcinoma harboring MET exon 14 skipping mutation: A case report. Front Oncol, 2022, 12: 954886. doi: 10.3389/fonc.2022.954886 36052259 PMC9424904

